# Co-Occurrence of High-Risk Human Papillomavirus and Herpesviruses Infections in Female Kidney Transplant Recipients: A Prospective One-Year Study

**DOI:** 10.3390/medicina62010149

**Published:** 2026-01-12

**Authors:** Maksims Cistjakovs, Liba Sokolovska, Baiba Lesina-Korne, Modra Murovska, Ieva Ziedina, Katerina Todorova, Alina Sultanova

**Affiliations:** 1 Institute of Microbiology and Virology, Riga Stradins University, LV-1067 Riga, Latvia; liba.sokolovska@rsu.lv (L.S.); modra.murovska@rsu.lv (M.M.); alina.sultanova@rsu.lv (A.S.); 2 Gynecology and Maternity Unit, Pauls Stradins Clinical University Hospital, LV-1002 Riga, Latvia; 3 Nephrology Department, Pauls Stradins Clinical University Hospital, LV-1002 Riga, Latvia; ieva.ziedina@rsu.lv; 4 Institute of Experimental Morphology, Pathology and Anthropology with Museum, Bulgarian Academy of Sciences, 1113 Sofia, Bulgaria

**Keywords:** human papillomaviruses, human herpesviruses, coinfection, kidney transplant recipients

## Abstract

*Background and Objectives*: Kidney transplant recipients (KTRs) face increased susceptibility to persistent viral infections due to prolonged immunosuppression. While high-risk human papillomavirus (HR-HPV) infection is known to be more prevalent in this population, little is known about the co-occurrence of HPV with human herpesviruses (HHVs) infection in the female genital tract. This study aimed to investigate the presence, dynamics, and potential interactions between HR-HPV and HHVs infections—including HSV-1, HSV-2, EBV, CMV, HHV-6, and HHV-7—in female KTRs during the first year after transplantation. *Materials and Methods*: A total of 39 female KTRs and 79 age-matched healthy controls were enrolled in the study. Cervicovaginal swabs from recipients were obtained at three time points: two weeks, six months, and one year post-transplantation. HPV DNA was screened using PCR, followed by high-risk HPV genotyping and quantitative viral load assessment using two commercial PCR kits. HHVs were detected using a multiplex PCR assay. *Results*: HPV DNA was detected in 98% of the KTRs at least once during follow-up, which was significantly greater than in the controls (38%). HR-HPV was identified in 46% of the recipients over the study period, with the highest viral load at one year post-transplantation. HHVs were detected in 72% of the KTRs but not in 43% of the controls (*p* < 0.01), with EBV and CMV being the most common. Coinfection with HR-HPV and HHVs occurred in 46% of the recipients but not in the controls. Samples containing both EBV and CMV had significantly higher HR-HPV viral loads than samples with no HHVs or with single/other HHV combinations (*p* < 0.01). All cervical intraepithelial neoplasia patients were found to have combined HPV and HHV infection. *Conclusions*: Female KTRs present a high burden of both HR-HPV and herpesviruses infections, with increased HPV viral loads. These findings suggest a potential synergistic interaction between HR-HPV and herpesviruses in the immunosuppressed setting.

## 1. Introduction

Kidney transplant recipients (KTRs) are at increased risk of persistent viral infections due to long-term immunosuppressive therapy. Among these, infection with high-risk human papillomavirus (HR-HPV) represents a particular concern, as impaired immune surveillance promotes viral persistence and increases the risk of cervical dysplasia and cancer. Previous studies and meta-analyses have consistently reported a higher prevalence of HR-HPV in KTRs than in the general population [1]. According to a study from the ERA register (2010–2018), the use of kidney transplantation (KT) has increased [2]. An increase of 14.3% in KT was observed in 2021 compared with 2020, which could explain the recent increase in the prevalence of HR-HPV among KTRs. This suggests that, if the trend continues, the prevalence of HPV infections among KTRs may increase further, despite the implementation of vaccination programs.

Several factors could contribute to the elevated HPV prevalence in KTRs, such as immunosuppression and HPV vaccination status. First, immunosuppression resulting from posttransplant therapy can be considered a major factor contributing to HPV persistence and, consequently, an elevated risk of cervical cancer development. Second, overall vaccine coverage remains low worldwide, and the median age of recipients means that they were most likely out of the target age once national HPV vaccination campaigns were implemented [1,3,4].

While immunosuppression is a key determinant of HPV persistence, interactions with other viruses may further influence HPV infection dynamics. Human herpesviruses (HHVs), including herpes simplex viruses (HSV-1/2), Epstein–Barr virus (EBV), cytomegalovirus (CMV), and human herpesviruses 6 and 7 (HHV-6/7), frequently establish latent infections and may reactivate under immunosuppressed conditions. Several studies in immunocompetent populations have reported codetection of HPV and HHVs in cervical samples, suggesting potential biological interactions within the genital mucosa [5,6,7,8,9,10]. Some studies even indicate that CMV and EBV may act as cofactors in HPV-16 oncogenesis [8]. To date, no studies have explored the coinfection of HPV and HHVs in KTRs.

Therefore, the aim of this prospective study was to evaluate the prevalence and co-occurrence of HR-HPV and selected human herpesviruses (HSV-1, HSV-2, EBV, CMV, VZV, HHV-6, and HHV-7) in cervical samples from female kidney transplant recipients during the first year after transplantation and to assess their relationship with the HPV viral load.

## 2. Materials and Methods

This prospective observational study was conducted at Pauls Stradiņš Clinical University Hospital and Riga Stradiņš University, Riga, Latvia. Female kidney transplant recipients who underwent transplantation between 2013 and 2015 were eligible for enrollment. Ethical approval was obtained from the Ethics Committee of Riga Stradiņš University (Approval No. E-9 [2] 20 May 2013). All participants provided written informed consent prior to inclusion. Female kidney transplant recipients aged ≥ 18 years who had undergone kidney transplantation during the study period and agreed to participate were eligible for inclusion. The exclusion criteria included prior hysterectomy, known gynecological malignancy at enrollment, pregnancy, acute graft rejection at the time of sampling, or inability to provide informed consent.

The control group consisted of immunocompetent women who underwent routine gynecological examinations and had no history of solid-organ transplantation, immunosuppressive therapy, or known cervical malignancy.

A total of 39 female KTRs (median age: 48 years; interquartile range [IQR]: 38–57) who underwent KT between 2013 and 2015 were enrolled in this study. The control group consisted of 79 healthy female individuals (median age: 48 years; IQR: 42–57) undergoing routine gynecological examinations.

The control group was intentionally larger than the transplant cohort to provide a stable reference population and improve the precision of prevalence estimates, given the limited number of eligible female kidney transplant recipients during the study period.

The most common underlying causes of end-stage renal disease among transplant recipients were chronic interstitial nephritis (26%), polycystic kidney disease (26%), hypertensive nephropathy (21%), and chronic glomerulonephritis (7%).

### 2.1. Sample Collection and Follow-Up

Cervical swab samples were collected prospectively at predefined time points (two weeks, six months, and one year post-transplantation) and processed for DNA extraction at the time of collection. The extracted DNA was stored under appropriate conditions until further analysis. While HPV testing was performed shortly after sample collection, comprehensive human herpesvirus (HHV) analyses were conducted retrospectively. At the time of study initiation, investigation of HHVs was planned; however, these analyses could not be completed immediately due to limited funding availability. Subsequent acquisition of additional financial support through the project “RSU internal and RSU with LSPA external consolidation,” aimed at supporting PhD research, enabled completion of the HHV analyses at a later stage using the stored DNA samples.

Cervical cytology was performed as part of routine gynecological follow-up via liquid-based cytology. The cytological findings were classified according to the Bethesda system. In cases of abnormal cytology (ASC-US or higher), patients were referred for colposcopic examination. Cervical intraepithelial neoplasia (CIN) was diagnosed on the basis of histological evaluation of colposcopy-guided cervical biopsy samples and was graded as CIN1, CIN2, or CIN3 according to standard pathological criteria.

### 2.2. Immunosuppressive Therapy

All renal transplant recipients received induction immunosuppressive therapy, which included a steroid bolus combined with monoclonal or polyclonal antibodies, as described in a previous study [11]. Maintenance immunosuppression consisted of the following:

Glucocorticoids: Prednisolone was tapered to 5 mg per day over the course of the study.

The antiproliferative agent mycophenolate mofetil (CellCept^®^, Roche Pharma AG, Grenzach-Wyhlen, Germany) was administered at 2 g per day and reduced to 1 g per day in cases of leukopenia.

Calcineurin inhibitors were administered either as once-daily tacrolimus (target trough concentrations: 7–10 ng/mL during the first three posttransplant months, followed by 5–8 ng/mL thereafter) or as microemulsified cyclosporine (target trough concentrations: 150–250 ng/mL for the first three months and 100–200 ng/mL in patients transplanted in 2013).

### 2.3. Viral Detection

Cervical swab samples were collected using sterile cytobrushes and immediately placed into transport medium. DNA extraction was performed via the standard chloroform–phenol method. DNA concentration and purity were assessed spectrophotometrically, and sample adequacy was confirmed by amplification of a human β-globin gene fragment. Initial screening for HPV DNA was carried out using the MY9/MY11 consensus primers, which target the L1 region of the HPV genome, as previously described [11].

Samples positive by consensus PCR were subsequently analyzed for high-risk HPV (HR-HPV) genotypes and viral load using two commercial real-time PCR assays. The quantitative detection of 12 HR-HPV types (16, 18, 31, 33, 35, 39, 45, 51, 52, 56, 58, and 59) was performed with the HPV High Risk Screen Real-TM Quant Kit (Sacace, Como, Italy) following the manufacturer’s protocol. HPV genotyping and semiquantitative viral load assessment were additionally performed using the Anyplex™ II HPV28 detection system (Seegene, Seoul, Republic of Korea).

Detection of human herpesviruses (HSV-1, HSV-2, EBV, CMV, HHV-6, and HHV-7) was carried out using the Allplex™ Meningitis-V1 multiplex PCR assay (Seegene, Republic of Korea), which enables simultaneous qualitative detection of multiple viral targets. All PCRs were run on a real-time PCR platform CFX90 C1000 Touch Thermal Cycler (Bio-Rad, Hercules, CA, USA), with appropriate positive and negative controls included in each run. Commercial kits were used strictly according to the manufacturers’ instructions.

### 2.4. Statistics

GraphPad Prism version 10.0 for Windows (GraphPad Software, San Diego, CA, USA) was used for all the statistical analyses and figure generation. Data normality was evaluated with the D’Agostino–Pearson, Anderson–Darling, and Shapiro–Wilk tests. Fisher’s exact test was used to compare the prevalence of HPV and HHV infection, while differences in viral load were analyzed with the Mann–Whitney U test. Because the majority of variables were not normally distributed, the data are presented as medians with interquartile ranges (IQRs). A *p* value < 0.05 was considered to indicate statistical significance.

## 3. Results

### 3.1. Detection of HPV Sequences Using MY9/11 Consensus Primers

Throughout the entire one-year follow-up period, HPV DNA was detected at least once in 38 out of 39 kidney transplant recipients (98%). This cumulative prevalence reflects positivity at any of the three posttransplant sampling time points.

At the first sampling point (two weeks post-transplantation), HPV DNA was detected in 27 recipients. Among these, 23 remained HPV positive at the six-month follow-up, whereas an additional 8 recipients who were HPV negative at two weeks became HPV positive at six months, resulting in a total of 31 HPV-positive individuals at that time point. At one year post-transplantation, HPV DNA was detected in 29 recipients.

Thus, although the number of HPV-positive individuals at individual time points ranged from 27–31, nearly all recipients (38/39) tested positive for HPV DNA at least once during follow-up, indicating a very high cumulative prevalence.

**Figure 1:**
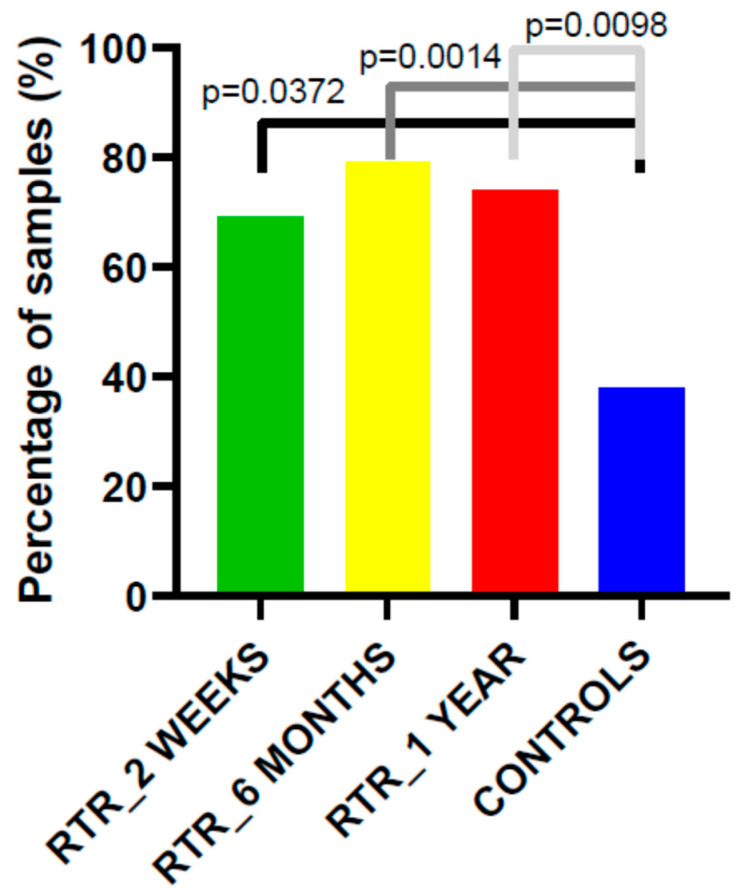
Prevalence of HPV DNA in kidney transplant recipients’ cervical samples at each follow-up time point using PCR with consensus primers. Statistical significance was assessed using the chi-square test.

In comparison, the frequency of HPV DNA detection in the control group was significantly lower—38% (30 out of 79)—than that in the recipient group at all measured time points: 69% at two weeks, 79% at six months, and 74% at one year post-transplantation (*p* < 0.05; Figure 1).

### 3.2. Detection of High-Risk HPV (HR-HPV) Sequences Using Two Commercial Real-Time PCR Kits

All samples that tested positive for HPV were further analyzed for HR-HPV types and viral load level.

During the entire one-year follow-up period, HR-HPV DNA was detected at least once in 18 out of 39 kidney transplant recipients, corresponding to a cumulative prevalence of 46%.

**Figure 2:**
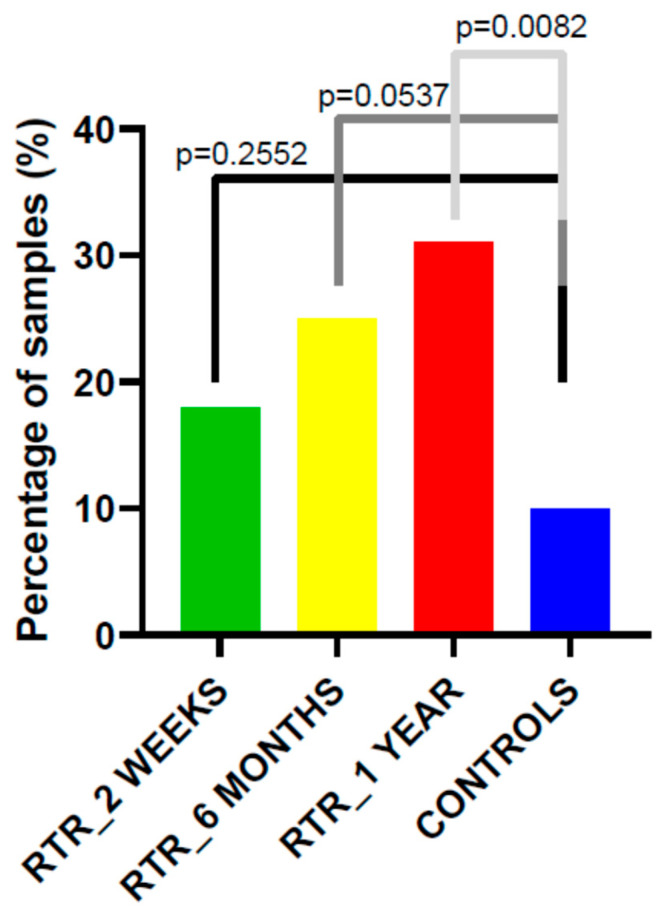
Prevalence of HR-HPV DNA in kidney transplant recipients’ cervical samples at each follow-up time point using Anyplex™ II HPV28 Detection system. Statistical significance was assessed using the chi-square test.

When analyzed by individual time points, HR-HPV DNA was detected in 7 recipients (18%) at two weeks post-transplantation. The proportion of HR-HPV–positive recipients increased to 25% (10/39) at six months and to 31% (12/39) at one-year post-transplantation. Thus, although the highest prevalence at a single time point was 31%, nearly half of the recipients were HR-HPV–positive at least once during follow-up. In contrast, only HR-HPV DNA was found only in 10% (8/79) of controls’ samples, although, significance was found only in comparison with RTR recipients after one-year time point (*p* = 0.0082, Figure 2).

The highest median HR-HPV viral load was observed one-year after transplantation, measuring 2.66 log copies per 10^5^ cells (IQR: 1.55–5.07). This was notably higher compared to the two-week period, which showed a median of 1.56 log copies per 10^5^ cells (IQR: 1.11–2.57), and the six-months mark, with a median of 2.51 log copies per 10^5^ cells (IQR: 1.64–6.58).

Among kidney transplant recipients, the following HR-HPV types were detected: 16, 18, 31, 33, 35, 51, 56, 66, 68, and 73. The most frequently identified type was HPV-18, found in 6 out of 39 recipients (15%), followed by HPV-16 (4/39; 10%), HPV-56 (4/39; 10%), HPV-35 (4/39; 10%), HPV-68 (3/39; 8%), HPV-51 (3/39; 8%), HPV-33 (2/39; 5%), HPV-31 (1/39; 2%), HPV-66 (1/39; 2%), and HPV-73 (1/39; 2%). Despite these variations, no statistically significant differences in type prevalence were observed.

HR-HPV infection was identified in 8% of individuals (7/79) in control group, a significantly lower prevalence compared with KTR, among whom 46% (18/39) tested positive (*p* < 0.01). A higher frequency of HR-HPV infection was also observed in KTR at all post-transplant time points: 18% at 2 weeks (*p* = 0.22), 25% at 6 months (*p* = 0.02), and 31% at 1 year (*p* < 0.01).

Although the median HR-HPV viral load in the control group (2.01 log copies per 10^5^ cells, IQR: 0.88–4.43) was higher than that observed in recipients two weeks after transplantation (1.56 log copies per 10^5^ cells, IQR: 1.11–2.57), this difference was not statistically significant. Among the controls, HR-HPV 16, 31, 33, 45, and 59 types were identified.

### 3.3. Determination of Human Herpesvirus Frequency Using Multiplex Detection Kit

**Figure 3:**
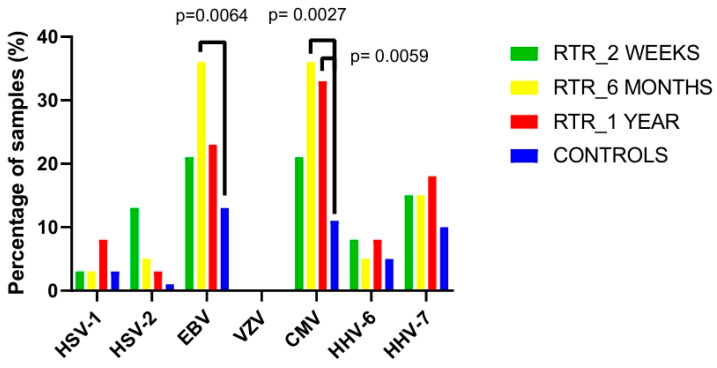
Numbers of different HHV DNA-positive samples among renal transplant recipients (RTRs) and controls at several time points. Statistical significance was assessed using the chi-square test.

Overall, 28 out of 39 (72%) recipients were positive for one or several HHV DNAs in their cervical swabs, which was a significantly greater percentage than that in the control group (34/79 [43%], *p* < 0.01). HSV-1, HSV-2, EBV, CMV, HHV-6 and HHV-7 DNA was detected in both groups. None of the individuals were positive for VZV DNA in the cervical swab samples. Among all herpesviruses, EBV and CMV were the most frequently found in both groups (for recipients after 6 months of surgery, 36% for both viruses and for controls, 13% were positive for EBV and 11% for CMV; Figure 1). Moreover, there was a significant difference in the frequency of the distribution of these two viruses in recipients after six months compared with that in controls (*p* = 0.0064 for EBV and *p* = 0.0027 for CMV). Notably, a significant difference remained for CMV in recipients one year after transplantation (*p* = 0.0059; Figure 3).

Although the frequency of HSV-2 was low in both groups, there was a significant difference between recipients after two weeks of transplantation and controls (13% [5/39] vs. 1% [1/79], respectively; *p* = 0.0148; Figure 3).

The frequencies of other herpesviruses did not significantly differ.

### 3.4. HPV and HHV Coinfection

The analyses presented in this section are based on cumulative data across the one-year follow-up period.

Among the recipients, 18 out of 39 (46%) were positive for both HR-HPV and HHV DNA in their cervical swabs. In contrast, none of the controls were positive for simultaneous HR-HPV and HHV infection.

**Figure 4:**
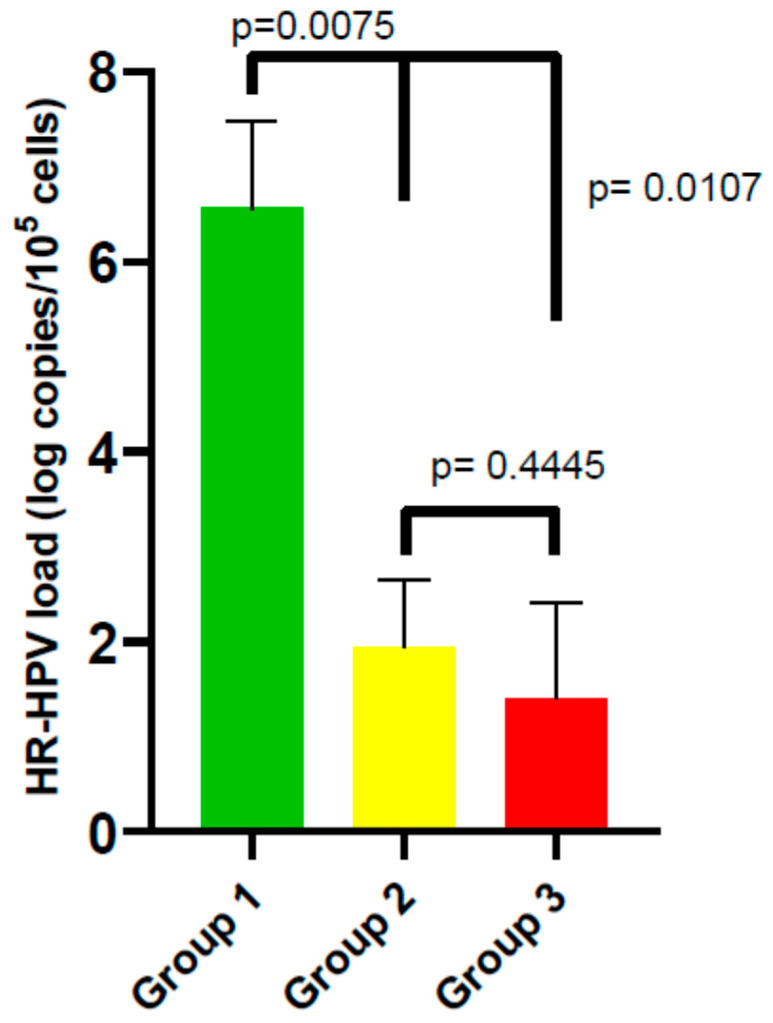
High-risk human papillomavirus load depending on the presence of HHV coinfection (Group 1: HR-HPV + EBV + CMV; Group 2: HR-HPV + other HHV(s); Group 3: HR-HPV alone). Statistical comparisons were performed using the Mann–Whitney U test.

HR-HPV viral load was significantly (using the Mann–Whitney U test) greater in samples coinfected with both EBV and CMV than in samples containing HR-HPV together with other HHVs (including HSV-1, HSV-2, HHV-6, or HHV-7, either alone or in combination with EBV + CMV) (median 6.56 [IQR: 2.53–7.48] vs. 1.95 [IQR: 1.16–2.66] log copies/10^5^ cells; *p* = 0.0075; Figure 4).

Additionally, the HPV load was found to be lower in the cervical samples without HHV DNA. Compared with samples containing CMV + EBV, a significant difference was found (median 1.400 [IQR: 0.375–2.405] vs. 6.56 [IQR: 2.53–7.48]; *p* = 0.0107); in turn, the difference was nonsignificant in comparison with samples containing single or different HHV coinfections (median 1.400 [IQR: 0.375–2.405] vs. median 1.95 [IQR: 1.16–2.66] log copies/10^5^ cells; *p* = 0.4445; Figure 4).

In addition, the histology results revealed that only six samples were positive for the presence of cervical intraepithelial neoplasia (CIN), and all these samples contained HPV + HHVs DNAs (where one of the viruses—CMV or EBV—was detected).

## 4. Discussion

In this prospective study of female KTRs, we found a markedly greater burden of human herpesviruses overall than in healthy controls, with EBV and CMV being the most frequently detected herpesviruses in the cervical samples of recipients (36% for both at six months) and a significantly greater frequency of EBV and CMV in recipients than in controls (*p* = 0.0064 and *p* = 0.0027, respectively). These findings were accompanied by frequent codetection of HR-HPV and herpesviruses: in nearly half (46%) of the recipients, the presence of simultaneous HR-HPV and HHV DNA was detected, and samples positive for both EBV and CMV had substantially higher HR-HPV viral loads than samples without these herpesviruses (median 6.56 vs. 1.40 log copies/10^5^ cells; *p* = 0.0075).

The disproportionate detection of EBV and CMV in the cervical samples of KTRs aligns with the well-established tendency of these viruses to reactivate under immunosuppression, leading to increased rates of viral replication and shedding in transplant recipient populations. Posttransplant reactivation of EBV and CMV is frequently reported in both the blood and tissue compartments of solid-organ transplant recipients and is associated with clinically important outcomes, including CMV disease and EBV-driven posttransplant lymphoproliferative disorder (PTLD) [12]. Several recent studies and reviews have documented frequent EBV and CMV replication in transplant recipient cohorts and emphasized overlapping episodes of EBV and CMV activation after transplantation [12,13].

Beyond systemic reactivation, the genital mucosa may be a permissive niche for EBV and CMV replication: population studies in nontransplanted women have documented detectable CMV and EBV DNA in cervical/urogenital samples, and work from several groups has shown increased cervical CMV/EBV detection in women with other genital infections or immunosuppression. These findings support the biological plausibility that immunosuppressed kidney recipients have both more frequent and higher-level shedding of CMV and EBV at the genital mucosa [14,15].

Nevertheless, several studies have reported the detection of VZV DNA in genital specimens at a low frequency, although genital involvement is uncommon for this viral infection [16,17]. Consistent with these findings, our study demonstrated that none of the cervical swab samples contained detectable VZV DNA.

Our observation that HR-HPV loads were significantly greater in samples copositive for EBV + CMV than in those positive for either HPV alone or HPV with other HHV combinations mirrors the accumulating evidence that certain herpesviruses may act as cofactors for HPV persistence and progression. Mechanistic and clinical studies indicate that EBV (and to a lesser extent CMV) can modulate epithelial cell biology and immune responses in ways that could promote HPV persistence or increase viral replication; several recent reviews and studies report frequent HPV–EBV co-occurrence in cervical lesions and suggest a possible synergistic role in oncogenesis. The finding that all biopsies with CIN in our cohort were HPV+ plus an HHV (commonly EBV or CMV) further supports a potential biological interaction worthy of prospective mechanistic studies [18,19,20].

Clinically, the higher HR-HPV loads observed with EBV + CMV codetection may help explain the elevated HR-HPV prevalence and persistence observed in transplant recipients compared with controls in our study and in prior meta-analyses of KTRs. Immunosuppression likely both increases primary susceptibility and reduces the host’s ability to clear HPV; concurrent herpesvirus reactivation could further promote viral persistence and, therefore, the risk of progression [21].

In contrast to EBV/CMV, HSV-2 DNA was infrequently detected in cervical samples, both in controls and at most time points among recipients—a pattern that has been observed previously. Population and cervical screening studies commonly show that, while HSV seroprevalence is high, active genital shedding (detectable viral sequence in mucosal DNA) is intermittent and may be relatively infrequent in asymptomatic women; when present, HSV tends to be episodic and often latent rather than persistently detectable in the cervical epithelium. In addition, HSV shedding is often episodic and confined to vesicular lesions or perilesional mucosa rather than being continuously detectable in cervicovaginal swabs, which can explain its lower detection rate than those of EBV and CMV in cross-sectional or periodic sampling. Several large studies of genital herpesvirus detection in general-population cervical samples reported very low detection rates for HSV-1 and HSV-2 DNA (often ~1–2%), concordant with our control group and with recent cervical viral prevalence studies [19,22].

This study has several limitations that temper causal inference. The sample size was modest and derived from a single center, which limits the statistical power and generalizability of the findings. Sampling was performed at predefined time points; therefore, intermittent viral shedding may have been missed, particularly for episodic infections such as HSV-2. In addition, HPV and human herpesvirus serological testing was not performed, precluding differentiation between primary infection, viral reactivation, or donor-derived infection, especially for latent viruses such as EBV and CMV. Systematic data on antiviral prophylaxis or treatment (e.g., valganciclovir use), which can profoundly affect CMV and EBV replication and detection, were also not available for analysis. Although immunosuppressive regimens and drug classes were documented, detailed longitudinal measures of immunosuppression intensity—such as drug trough levels, cumulative exposure, or immune function parameters at each sampling point—were not collected. Finally, while associations between EBV/CMV codetection, higher HR-HPV viral load, and cervical intraepithelial neoplasia were observed, the present data cannot be used to determine whether EBV or CMV causally promotes HPV persistence or instead reflects impaired local immune control. Future studies should include larger, multicenter cohorts with paired serology, quantitative longitudinal viral measurements, standardized antiviral prophylaxis documentation, and detailed immunosuppression profiling to better clarify the biological and clinical significance of HPV–herpesvirus interactions in kidney transplant recipients.

In female KTRs, EBV and CMV are disproportionately present in cervical samples compared with healthy controls, and their joint presence with HR-HPV is associated with higher HPV loads and with the limited number of CIN cases observed in this studied group. HSV-2 DNA is infrequently detected, likely reflecting episodic shedding and the biology of HSV latency rather than the absence of prior exposure. Our findings align with recent literature describing frequent EBV/CMV replication in transplant recipients and mounting evidence that EBVs (and possibly CMV) may act as cofactors in HPV persistence and progression. Prospective studies are needed to test whether interventions that reduce EBV–CMV activation (for example, modification of immunosuppressive regimens or targeted prophylaxis) can reduce HR–HPV persistence and the risk of cervical disease in transplant recipients.

## 5. Conclusions

In this prospective one-year study of female KTRs, we demonstrated a substantially greater prevalence of both high-risk human papillomavirus and human herpesvirus infections than did healthy controls. EBV and CMV were the most frequently detected herpesviruses in the cervicovaginal samples of recipients, and their occurrence remained significantly elevated beyond the early posttransplant period. Nearly half of the recipients exhibited simultaneous HR-HPV and HHV infection, a pattern not observed in any control participants.

Coinfection with EBV and CMV was strongly associated with a markedly increased HR–HPV load, suggesting that these herpesviruses have a potent effect on HPV persistence or replication.

These findings highlight a substantial viral burden in female KTRs and suggest that EBV and CMV reactivation may influence the course of HR-HPV infection. Larger and longer-term studies are needed to determine whether targeted strategies to reduce herpesvirus activity or adjust immunosuppression could mitigate HR–HPV persistence and lower the risk of cervical disease in this high-risk population.

## Data Availability

The data that support the findings of this study are available from the corresponding author upon reasonable request.
